# Supplementary material for: Oxytocin receptor DNA methylation is associated with exogenous oxytocin needs during parturition and postpartum hemorrhage
Source: Commun Med (Lond). 2023 Jan 27;3:11. doi: 10.1038/s43856-023-00244-6 (PMC9882749; doi:10.1038/s43856-023-00244-6)
Supplement: Supplementary file 3 — Description of Additional Supplementary Files [file 43856_2023_244_MOESM3_ESM.pdf]

Description of Additional Supplementary Files

File Name: Supplementary Data 1

Description: Supplementary file with source data for figures 2-4
