# Supplementary material for: Oxytocin receptor DNA methylation is associated with exogenous oxytocin needs during parturition and postpartum hemorrhage
Source: Commun Med (Lond). 2023 Jan 27;3:11. doi: 10.1038/s43856-023-00244-6 (PMC9882749; doi:10.1038/s43856-023-00244-6)
Supplement: Supplementary file 4 — Reporting Summary [file 43856_2023_244_MOESM4_ESM.pdf]

nature research

Corresponding author(s): Elise N Erickson, PhD CNM

Last updated by author(s): Dec 13, 2022

Reporting Summary

Nature Research wishes to improve the reproducibility of the work that we publish. This form provides structure for consistency and transparency in reporting. For further information on Nature Research policies, see our Editorial Policies and the Editorial Policy Checklist.

Statistics

For all statistical analyses, confirm that the following items are present in the figure legend, table legend, main text, or Methods section.

n/a	Confirmed	
☐	☒	The exact sample size (*n*) for each experimental group/condition, given as a discrete number and unit of measurement
☐	☒	A statement on whether measurements were taken from distinct samples or whether the same sample was measured repeatedly
☐	☒	The statistical test(s) used AND whether they are one- or two-sided<br>*Only common tests should be described solely by name; describe more complex techniques in the Methods section.*
☐	☒	A description of all covariates tested
☐	☒	A description of any assumptions or corrections, such as tests of normality and adjustment for multiple comparisons
☐	☒	A full description of the statistical parameters including central tendency (e.g. means) or other basic estimates (e.g. regression coefficient) AND variation (e.g. standard deviation) or associated estimates of uncertainty (e.g. confidence intervals)
☐	☒	For null hypothesis testing, the test statistic (e.g. *F*, *t*, *r*) with confidence intervals, effect sizes, degrees of freedom and *P* value noted<br>*Give P values as exact values whenever suitable.*
☒	☐	For Bayesian analysis, information on the choice of priors and Markov chain Monte Carlo settings
☒	☐	For hierarchical and complex designs, identification of the appropriate level for tests and full reporting of outcomes
☒	☐	Estimates of effect sizes (e.g. Cohen's *d*, Pearson's *r*), indicating how they were calculated

Our web collection on statistics for biologists contains articles on many of the points above.

Software and code

Policy information about availability of computer code

Data collection	REDCap was used for clinical and participant level data collection
Data analysis	Stata SE (17.0), R packages (minifi, noob, shinyMethyl, ewastools). Cross tissue analysis: Stata SE(17.0), R (ggplot2, dplyr,haven, ggsci, modelr,tidyverse)

For manuscripts utilizing custom algorithms or software that are central to the research but not yet described in published literature, software must be made available to editors and reviewers. We strongly encourage code deposition in a community repository (e.g. GitHub). See the Nature Research guidelines for submitting code & software for further information.

Data

Policy information about availability of data

All manuscripts must include a data availability statement. This statement should provide the following information, where applicable:

- Accession codes, unique identifiers, or web links for publicly available datasets
- A list of figures that have associated raw data
- A description of any restrictions on data availability

Data and materials availability: Data, code, and materials used in the analyses can be available for purposes of reproducing or extending the analyses in collaboration with the lead author. Materials transfer agreements (MTAs) may be required. Extensions of biological work may be limited to participants' data who consented to specimen use in future research.

Field-specific reporting

Please select the one below that is the best fit for your research. If you are not sure, read the appropriate sections before making your selection.

☒ Life sciences ☐ Behavioural & social sciences ☐ Ecological, evolutionary & environmental sciences

For a reference copy of the document with all sections, see nature.com/documents/nr-reporting-summary-flat.pdf

Life sciences study design

All studies must disclose on these points even when the disclosure is negative.

Sample size	Tissue match analysis: A sample of 19 women will be sought for this study based on an expected moderate correlation of (0.6), alpha 0.05 and power of 80%. Case-control: Sample Size Justification: With a sample size of 200 (100 women per group), we will have 80% power to detect a mean difference in methylation of 0.2 (%), (based on level of significance of 0.05 and standard deviation of 1.3). (this sample size was not achieved due to pandemic limitations.)
Data exclusions	Specimens excluded are detailed due to quality control
Replication	Pyrosequencing was performed in triplicate to ensure reliable DNA methylation findings. Replicate variability was used as a control in the analysis as well.
Randomization	The covariates of parity and body mass index were used in regression models as they significantly differed between cases and controls and predicted oxytocin use independently. Replicate variability was also used as a control in the models. Antibiotic administration was included as a control as it differed between cases/controls.
Blinding	The collaborators who perfomed the bench experiments and pyrosequencing were blinded to the case/control status

Reporting for specific materials, systems and methods

We require information from authors about some types of materials, experimental systems and methods used in many studies. Here, indicate whether each material, system or method listed is relevant to your study. If you are not sure if a list item applies to your research, read the appropriate section before selecting a response.

Materials & experimental systems

n/a	Involved in the study	
☒	☐	Antibodies
☒	☐	Eukaryotic cell lines
☒	☐	Palaeontology and archaeology
☒	☐	Animals and other organisms
☐	☒	Human research participants
☐	☒	Clinical data
☒	☐	Dual use research of concern

Methods

n/a	Involved in the study	
☒	☐	ChIP-seq
☒	☐	Flow cytometry
☒	☐	MRI-based neuroimaging

Human research participants

Policy information about studies involving human research participants

Population characteristics	The enrolled sample consisted of 69 postpartum hemorrhage cases and 50 control participants. Similarities between groups included maternal age (mean 31 vs 32 years) , gestational age (mean 39 for both) , presence of antenatal complications (hypertension/diabetes), and ethnic background/ancestry (78%, 79% white/european ancestry-- however Hispanic participants were more frequent in the case group (15.9% vs. 2 % of controls).
Recruitment	Participants were invited to participate who gave birth in the greater Portland, Oregon area including hospitals and community birth settings (home/birth center). Those giving birth at our institution were given information about the study in the postpartum unit or at a follow-up visit based on meeting basic screening criteria in the electronic health record. Other participants responded to advertisements posted in the community and on social media.<br><br>We translated all of our materials into Spanish and were able to enroll using an interpreter service, however, only one Spanish-speaking person who was approached enrolled in the study. We do not know if this was due to not having a billingual recruiting staff member or if there were other reasons for this. The greater Portland area is not known for great diversity, particularly for African American individuals- thus the sample is biased in towards the community demographics.

Ethics oversight | Oregon Health and Science University

Note that full information on the approval of the study protocol must also be provided in the manuscript.

Clinical data

Policy information about clinical studies

All manuscripts should comply with the ICMJE guidelines for publication of clinical research and a completed CONSORT checklist must be included with all submissions.

Clinical trial registration	n/a
Study protocol	*Note where the full trial protocol can be accessed OR if not available, explain why.*
Data collection	*Describe the settings and locales of data collection, noting the time periods of recruitment and data collection.*
Outcomes	*Describe how you pre-defined primary and secondary outcome measures and how you assessed these measures.*
